# Oral cancer awareness and knowledge among patients attending an oncological screening program in Portugal

**DOI:** 10.4317/medoral.27081

**Published:** 2025-04-06

**Authors:** Margarida Andrade, Diogo Pereira, Beatriz Garcia, André Moreira, João Caramês, Marta Pojo, Filipe Freitas

**Affiliations:** 1 DDS. School of Dental Medicine, University of Lisbon, Lisbon, Portugal; 2 MSc. The Portuguese Cancer League, Lisbon, Portugal; 3 DDS, PhD. School of Dental Medicine, University of Lisbon, Lisbon, Portugal; 4 MSc, PhD. The Portuguese Cancer League, Lisbon, Portugal

## Abstract

**Background:**

The aim of this study was to evaluate the awareness and knowledge about oral cancer in a Portuguese population and to assess its possible relations with sociodemographic variables.

**Material and Methods:**

A cross-sectional questionnaire survey was conducted, with 2650 individuals that attended oncological screening initiatives in the southern region of Portugal. The questionnaire collected data on participants’ socio-demographic information, life-style habits, and awareness of oral cancer.

**Results:**

Over four-fifths of the participants (83.4%) mentioned that had already heard about oral cancer. Knowledge of oral cancer was significantly associated with age (*p* = 0.034), education level (*p* < 0.001), frequency of dentist visits (*p* < 0.001), and smoking (*p* = 0.015) and alcohol habits (*p* = 0.031). Regarding risk factors, smoking and alcohol usage were identified by 86% and 58.5% of the sample, respectively. Only 62.8%, 45.5%, and 37.9%, respectively, were able to correctly identify ulcers that don’t heal, red and white lesions, and lumps/tissue that overgrows as early signs of oral cancer.

**Conclusions:**

A general lack of awareness regarding oral cancer was found in this Portuguese population. Lower levels of knowledge were found in individuals of higher risk for oral cancer. Therefore, educational programs targeting the risk population are highly recommended for the primary prevention and early diagnosis of oral cancer in the Portuguese population.

** Key words:**Oral cancer, oral cancer screening, awareness, knowledge, literacy.

## Introduction

Oral cancer (OC) is a major public health problem around the globe, due to its high incidence, mortality, and morbidity rates (1-3). It is a malignant neoplasia which affects the oral cavity, that includes the lips, tongue, gingiva, mouth floor, and salivary glands (C00-C08) (4). Squamous cell carcinomas constitute approximately 90% of oral cancer cases (5).

According to Global Cancer Observatory (GCO), lip, oral cavity, and pharyngeal cancers accounted with about 747.316 new cases and 367.285 deaths in 2020 (6). In Portugal, according to the National Cancer Registry of 2019, OC was found to be the 6th most frequent cancer in men, with 1000 of the 1335 new cases reported.

OC mostly affects men, aged 40 to 70 years old (2-3). Oral cancer is a multifactorial lesion, with alcohol and tobacco being the most important risk factors. In fact, they seem to be present in 80-90% of cases (4,7). In addition, though they are independent risk factors, when combined, they present a significant synergetic effect (8). Other risk factors include papillomavirus infections, a poor diet in fruits and vegeTables, and sunlight exposure, which is the main etiological factor for the lower lip squamous cell carcinoma (9-11).

Oral cancer is easily diagnosed given the visual accessibility of the oral mucosa, being the margins of the tongue and the floor of the mouth, the anatomical sites more frequently affected (12). However, in Portugal, nearly 50% of cases are diagnosed in advanced stages of the disease (13). It is important to notice that the 5 year-survival rate is only 20% in advanced stages (stages III/IV), whereas in early diagnosed stages (stage I) is almost 80%, which translates the importance of the recognition of early signs and symptoms (6).

Furthermore, as most risk factors consist in lifestyle factors, oral cancer should be an easily prevenTable pathology (14). However, the lack of awareness and information of the population regarding this type of cancer and its risk factors explains the high mortality and morbidity rates. That is why international guidelines have addressed the importance of the literacy in this matter, to allow early detection and primary prevention, by the adoption of a healthy lifestyle (15).

The aim of this study was to analyze the level of awareness regarding oral cancer in a population monitored in an oncological screening in Portugal, and to evaluate its possible relations with sociodemographic variables and lifestyle.

## Material and Methods

A questionnaire-based survey was performed in the waiting areas of an oral cancer screening program, organized by the non-profit organization Liga Portuguesa Contra o Cancro - Núcleo Regional do Sul (LPCC-NRS).

Individuals over the age of 18 were interviewed in 16 local administrative units of the LPCC-NRS (Abrantes, Alcaìcer do Sal, Almodôvar, Beja, Borba, Eìvora, Faro, GolegaÞ, Meìrtola, Moura, Peniche, Portalegre, Santareìm, Seixal, Setuìbal e Torres Novas), between the 19th of March and the 17th of December 2022. During this time, forty-eight screening actions took place, where 3187 subjects were observed.

Out of the forty-eight sessions, only forty-two were included in the study, due to availability of the interviewers. Therefore, of the 3187 individuals, only 2650 were surveyed and included in the sample.

The primary target audience for LPCC-NRS consisted of males over 40 years old who smoked and consumed alcohol. However, the screenings were open to all, with no restrictions on participant criteria.

A questionnaire, specifically designed for this study, was implemented by previously trained and calibrated interviewers, who were last year students of the Dental Medicine graduation program in Lisbon. Authorization and ethical approval were previously obtained by LPCC. Participation in the study was voluntary and the answers were registered anonymously. A written consent was obtained for each participant prior to the realization of each interview. All participants were aware that it was possible to leave the study at any time.

The questionnaire consisted in two different parts: sociodemographic and lifestyle characterization; oral cancer awareness characterization and knowledge of the sampled population, where 16 questions were made.

Statistical analyses were carried out using IBM SPSS Statistics version 28.0 software (IBM Corporation, NY, USA). Qualitative data were presented in relative and absolute frequencies. Possible associations between OC awareness and sociodemographic and personal habits variables were analyzed by Chi-square test. However, the utilization of this test requires two assumptions: there should not be more than 20% of cells in the Table with expected values less than 5, and the minimum expected value should be greater than 1. So, in cases where these assumptions were not verified, it was used the alternative test, the Fischer-Freeman-Halton exact test. The significance level was set at *p* < 0.05.

## Results

A total of 2650 questionnaires were collected during these screening actions. The characterization of the participants relative to its sociodemographic data and lifestyle habits is summarized in Table 1 and in Table 2. Out of all participants, 804 (30.3%) were male and 1846 (69.7%) were female. The mean age was 57.33 years (SD ± 13.76), ranging from 18 to 92 years old. The great majority of individuals were Caucasian (n = 2638; 99.5%).

The awareness and knowledge about OC were assessed via close-ended and multiple-choice questions. Absolute and relative frequencies of the responses to all 16 questions are listed in Table 3. Most of the participants (83.4%) had already heard about oral cancer. However, only about a third (37.2%) considered OC as one of the most frequent cancers in Portugal.

In general, awareness was associated with lower age (*p* = 0.034), high levels of education (*p* 0.001), former smoking habits (*p* = 0.015), alcohol habits (*p* = 0.031) and regular dentist visits (*p* 0.001). All established associations between oral cancer knowledge and sociodemographic or lifestyle characteristics are presented in the Supplement 1.

Several questions were made to assess the knowledge of risk factors and susceptibility for oral cancer. More than 40% of the respondents were aware that OC is most frequent between the ages of 40 and 60 years old, while only 27.9% knew that the male gender is more affected in comparison with the female gender, as 42.8% of the participants answered both genders are equally affected. In this matter, participants over 49 years old, were less informed about its greater risk of having OC (*p* 0.001), and men were less aware of its susceptibility than women (*p* = 0.014).

Tobacco was identified by most individuals (86%) as a risk factor, whereas alcohol was pointed out by 58.5% of the inquired, following poor oral hygiene which was referred by 59.4%. Other factors mentioned were family history of cancer (41.5%), infections (34.6%), poor diet in fruits and vegeTables (23.1%), and exposure to sunlight (10%). The recognition of tobacco as a risk factor was associated with lower age (*p* 0.001), higher education (*p* 0.001), former smoking habits (*p* 0.001) and regular visits to the dentist (*p* 0.001). Alcohol was identified as a risk factor for oral cancer more often by individuals with higher education level (*p* 0.001) and with more than one visit per year to their dentist (*p* 0.001).

Among the respondents, 85.9% answered that oral cancer could be prevented, and 12.2% admitted to not knowing. Regular visits to the dentist and tobacco cessation were the two most mentioned forms of oral cancer prevention (70.5% and 66%, respectively).

Generally, a poor knowledge regarding the early signs and symptoms of OC was showed. Less than two thirds of the individuals (62.8%) identified an ulcer that doesn’t heal as a sign of oral cancer, and only 45.5% and 37.9% identified white or red spots and lump/tissue that overgrows, respectively. The awareness of ulcers that don’t heal and a white or red spots as signs of OC were demonstrated superior in females (*p* 0.001) and in individuals with higher education (*p* 0.001) and that visit the dentist more frequently (*p* < 0.001).

When questioned what the primary affected locations by OC were, 44.9% and 22.1% correctly answered the tongue and the floor of the mouth, respectively.

Regarding treatment options, almost half of the population (45.8%) revealed that they did not know the treatment possibilities. The knowledge of surgery as the ideal treatment option was superior in women (*p* = 0.002), younger individuals (*p* = 0.006), with higher education (*p* 0.001) and more frequent visits to the dentist (*p* = 0.022).

Nearly all participants (98%) answered that the detection of oral cancer in early stages is associated with a better prognosis. High education level was significantly related with this answer (*p* 0.001).

Additionally, 88.5% demonstrated awareness that oral cancer is potentially fatal. Older individuals and those with higher levels of education were found to be more informed (*p* = 0.016 and *p* < 0.001, respectively).

When asked whether their dentist screens them for signs of oral cancer more than 90% answered no or that they did not know. Also, dentists were referred as an OC source of information by only 12.2% of individuals, being the television or radio (37.3%) and the internet (33%) the most mentioned sources. The internet was more significantly mentioned by younger individuals (*p* 0.001).

Finally, 92.2% expressed an interest in learning more about OC.

## Discussion

To the best of the authors’ knowledge, this is the first study of this type to be made in Portugal. Two similar studies took place in the cities of Valongo and Porto, where questionnaires with the same purpose were conducted (7,13). However, they were administered in the context of dental consultations, being this the first Portuguese investigation that aimed to assess the knowledge and awareness about OC in the context of invitational OC screenings.

The percentage of participants who reported having heard of oral cancer before was 83.4%, similar to the 87.3% reported in Saudi Arabia, but higher than the ones registered in other previous studies (13,16-20). This difference may be related to the fact that these individuals attended the screenings consciously and voluntarily, which demonstrates a greater interest and concern for their health.

The predominance of female participants in the sample (69.7%) is in line with other reports and can be explained by random variation or a lesser interest among men in participating in the screenings. In fact, although males have a higher incidence rate for OC, it is proven that they tend to underutilize preventive healthcare services (19,21).

Tobacco was the most frequently acknowledged risk factor (86%). Alcohol, on the other hand, was mentioned by only 58.5% of respondents, as observed in other reports (17,20-22). Greater awareness of tobacco's contribution to the etiology of oral cancer may be attributed to anti-tobacco campaigns that highlight its adverse effects. However, the role of alcohol should be emphasized, both in its isolated effect and, more importantly, in its synergistic effect with tobacco.

Factors such as sunlight exposure and a poor diet in fruits and vegeTables were identified by significantly low percentages (10% and 23.1%, respectively), which, although consistent with some studies, did not match the findings of Al Hulaibi *et al* (1,16,17,19). However, when asked about prevention methods, participants identified a healthy diet as one of the main forms of prevention.

A relatively high percentage of participants (34.6%) identified infections as a risk factor, compared to studies where only 25% of individuals were able to point out this factor (23). It is essential to highlight the role of infections such as HPV, especially its subtypes 16 and 18, which, despite having a more prominent role in the etiology of oropharyngeal cancer, also seem to act on oral cavity cancer (24). Therefore, vaccination against HPV should also be promoted, as it was only mentioned as a prevention method by about 10% of the sample.

Lack of awareness of early and characteristic signs can lead to late diagnoses and have a fatal impact on the disease’s prognosis. In this sample, high percentages in the recognition of non-healing ulcers, red or white patches, and lump/tissue that overgrows were observed (62.8%, 45.5%, and 37.9%, respectively), with only about 22% of participants stating they couldn't identify any signs or symptoms among the provided options. Less positive results were observed in other investigations (13,15,16,20). This could be explained by the invitational nature of the screenings under study, as participants may exhibit one of the enumerated signs.

Regarding the available treatment options, almost half of the individuals (45.8%) admitted having no knowledge of them and only about a third could identify surgery, chemotherapy, and radiation therapy as treatment options (36.6%, 35.5%, 26.9%, respectively), which is much lower than other reports (16).

Most individuals (98%) believed that an early diagnosis results in a better treatment prognosis, which is in line with the results of studies in the United Kingdom and Portugal (13,22).

A very low percentage of participants, compared to other reports, claimed to have been screened for oral cancer by their dentist (9.1%) (24). Note that this study does not seek to determine whether dentists are in fact conducting these screenings or not. However, it indicates that patients are unaware if they are being screened for oral cancer, which can be considered both positive, as knowledge can generate unnecessary anxiety in patients, and negative. In fact, Choi *et al* founded that patients would like to know if they are being screened for OC (25).

The sources of information were in line with what is described in the literature, consisting mainly of media, specifically television and radio (37.3%) (1). The intern*et al*so proved to be an important mean of knowledge transmission (33%), especially among younger individuals, as expected (20). The fact that dentists were mentioned as a source of information by only 12.2% of participants is alarming, although the same has been observed in other investigations. It is essential to emphasize that non-medical sources, although increase basic knowledge and contribute to awareness of OC, may be insufficient, so the role of these professionals should not be overlooked (17).

It is important to notice that, as a cross-sectional study, some limitations and bias may be present. In fact, although this survey had one of the largest samples in Europe, it may not be representative of the entire Portuguese population. There is an inherent bias in the sample, as those who attended these screenings are inherently more interested and concerned about their health. In addition, it was made a geographical restriction not only to the southern region of Portugal, as to areas dislocated from city centers, as the aim of the LPCC-NRS was reaching isolated populations that do not have easy access to healthcare services.

Another limitation is the use of a multiple-choice questionnaire. By providing multiple options to participants, they may randomly answer without possessing a true knowledge on the matter. However, this was partly counteracted by administering the questionnaire through interviews rather than having participants fill it out in writing. Additionally, the fact that the answers were registered anonymously, may also have reduced this bias.

## Conclusions

Despite most of the participants mentioned that had already heard about oral cancer, a general lack of awareness was found in this Portuguese population, mainly regarding epidemiology, risk factors and clinical manifestations of oral cancer. Lower levels of knowledge were found in individuals of higher risk for oral cancer, namely men, aged over 40 years, with smoking and/or alcohol habits. Therefore, educational programs targeting the risk population are highly recommended for the primary prevention and early diagnosis of oral cancer in the Portuguese population. Further research is required to corroborate the results obtained, with samples more representative of the general population.

## Figures and Tables

**Table 1:** Sociodemographic and lifestyle characterization of the sampled population.

Characteristics		n	%
Sex	Male	804	30.3
	Female	1846	69.7
Age	≤ 49 years	716	27.0
	> 49 years	1934	73.0
Population affinity	Leucodermic	2638	99.5
	Melanodermic	11	0.4
	Xanthodermic	1	0.1
Education	No level of school	32	1.2
	Primary school	841	31.7
	High school	972	36.7
	Technical vocation/University	805	30.4
Tobaco comsumption	Non-smoker	1451	54.8
	Smoker	477	18.0
	Ex-smoker	722	27.2
Alcohol comsumption	Non-consumer	1145	43.2
	Consumer	1401	52.9
	Ex-consumer	104	3.9
Dentist visits	< 1x per year	1122	42.3
	≥ 1x per year	1528	57.7
Last dentistry appointment	Lats 6 months	1124	42.4
	6 months - 1 year	202	7.6
	1 - 2 years	497	18.8
	Over 2 years	608	22.9
	Do not recall	219	8.3

**Table 2:** Characterization of the smokers and alcohol consumers of the sampled population.

Characteristics			n	%
Smokersn = 477(18.0%)	Cigarettes per day	< 20 cig/day	361	75.7
		≥ 20 cig/day	116	24.3
	Habit duration	< 20 years	158	33.1
		≥ 20 years	319	66.9
	Type of cigarettes	Normal	438	91.8
		Electronic	27	5.7
		Both	12	2.5
Alcohol consumersn = 1401(52.9%)	Habit frequency	Diary	396	28.1
		Occasional: < 1day/week	607	43.5
		Occasional: 1 day/week	177	12.3
		Occasional: 2 days/week	135	9.6
		Occasional: 3 days/week	68	4.8
		Occasional: 4 days/week	13	0.9
		Occasional: 5 days/week	8	0.6
		Occasional: 6 days/week	4	0.3
	Habit duration	<20 years	398	28.3
		20 years	1008	71.5

**Table 3:** Answers to the questionnaire by the sampled population.

Questions		n	%
Before coming to this screening, had you already heard of oral cancer?	Yes	2209	83.4
	No	441	16.6
Do you think oral cancer is among the top 10 most common cancers in Portugal?	Yes	985	37.2
	No	439	16.6
	Don't know	1226	46.3
Which age group do you think is most affected by oral cancer?	0 to 19 years	4	0.2
	20 to 39 years	126	4.8
	40 to 59 years	1093	41.2
	60 or more years	554	20.9
	Don't know	873	32.9
What do you think is the gender most affected by oral cancer?	Females	129	4.9
	Males	739	27.9
	Both	1133	42.8
	Don't know	649	24.5
What factor(s) do you think can cause oral cancer?	Tobacco	2279	86
	Alcohol	1551	58.5
	Stress	487	18.4
	Bad oral hygiene	1575	59.4
	Family history of cancer	1100	41.5
	Sunlight exposure	264	10
	Ill-fitting dentures	559	21.1
	Poor diet in fruits and vegetables	612	23.1
	Infections	917	34.6
	None, it's spontaneous	35	1.3
	Don't know	172	6.5
Do you believe oral cancer can be prevented?	Yes	2276	85.9
	No	53	2.0
	Don't know	325	12.2
If you think it can be prevented, how do you think it can be prevented?	Tobacco cessation	1749	66.0
	Moderate alcohol consumption	1269	47.9
	Regular visits to the dentist	1869	70.5
	Sunlight protection	314	11.8
	Brushing teeth	1453	54.8
	Healthy diet	1131	42.7
	Vaccination	257	9.7
	Don't know	81	3.1
Did you know that there are pre-malignant lesions that can appear before cancer?	Yes	1565	59.1
	No	1085	40.9
What changes do you consider could be cancer/pre-malignant lesions?	Lump/tissue that overgrows	1004	37.9
	Red or white spot	1207	45.5
	Non-healing ulcer	1665	62.8
	Bleeding	944	35.6
	Tooth infection	784	29.6
	Reduced mouth opening	438	16.5
	Difficulty moving the tongue	881	33.2
	Pain when swallowing	837	31.6
	Frequent aphthous ulcers	635	24.0
	Don't know	577	21.8
In which area(s) of the mouth do you think the development of oral cancer is most frequent?	Lip	557	21.0
	Tongue	1190	44.9
	Buccal mucosa	349	13.2
	Palate	544	20.5
	Floor of the mouth	586	22.1
	Maxillary bones	358	13.5
	Don't know	829	31.3
What type(s) of treatment do you think correspond to the treatment of oral cancer?	Surgery	969	36.6
	Chemotherapy	941	35.5
	Radiotherapy	714	26.9
	Medication	397	15.0
	Don't know	1215	45.8
Do you think treatment has a higher success rate if the tumor is diagnosed early?	Yes	2596	98.0
	No	18	0.7
	Don't know	36	1.4
Do you think regular follow-up is necessary after oral cancer treatment?	Yes	2625	99.0
	No	10	0.4
	Don't know	15	0.6
Do you think oral cancer can be fatal?	Yes	2346	88.5
	No	172	6.5
	Don't know	132	5.0
Has your dentist ever conducted an evaluation/screening to check for oral cancer?	Yes	241	9.1
	No	2399	90.5
	Don't know	10	0.4
What source(s) of information did you use to gain knowledge about oral cancer?	Family or friends	870	32.8
	Doctor	289	10.9
	Dentist	323	12.2
	Internet	874	33.0
	Television or radio	989	37.3
	Newspaper or magazine	381	14.4
	Cigarette packs	278	10.5
	Awareness campaigns	437	16.5
	Others	157	6.0
	No source of information	242	9.1
Would you like to have more knowledge about oral cancer?	Yes	2442	92.2
	No	208	7.8
